# Apoptosis-Inducing Factor, Mitochondrion-Associated 3 (AIFM3) Protein Level in the Sera as a Prognostic Marker of Cholangiocarcinoma Patients

**DOI:** 10.3390/biom10071021

**Published:** 2020-07-10

**Authors:** Daraporn Chua-on, Tanakorn Proungvitaya, Doungdean Tummanatsakun, Anchalee Techasen, Temduang Limpaiboon, Sittiruk Roytrakul, Sopit Wongkham, Chaisiri Wongkham, Ongart Somintara, Sakkarn Sangkhamanon, Siriporn Proungvitaya

**Affiliations:** 1 Centre of Research and Development of Medical Diagnostic Laboratories, Faculty of Associated Medical Sciences, Khon Kaen University, Khon Kaen 40002, Thailand; chuaondaraporn@kkumail.com (D.C.); tanakorn@kku.ac.th (T.P.); pui_ddlab41@hotmail.com (D.T.); anchaleetechasen@gmail.com (A.T.); temduang@kku.ac.th (T.L.); 2 Cholangiocarcinoma Research Institute, Faculty of Medicine, Khon Kaen University, Khon Kaen 40002, Thailand; sopit@kku.ac.th (S.W.); chaisiri@kku.ac.th (C.W.); 3 National Center for Genetic Engineering and Biotechnology, National Science and Technology Development Agency, Pathumthani 12120, Thailand; sittiruk@biotec.or.th; 4 Department of Biochemistry, Faculty of Medicine, Khon Kaen University, Khon Kaen 40002, Thailand; 5 Department of Surgery, Faculty of Medicine, Khon Kaen University, Khon Kaen 40002, Thailand; ongaso@kku.ac.th; 6 Department of Pathology, Faculty of Medicine, Khon Kaen University, Khon Kaen 40002, Thailand; sakkarn@kku.ac.th

**Keywords:** AIFM3, serum, dot blot, prognostic marker, CCA

## Abstract

Prognosis of cholangiocarcinoma (CCA) patients is absolutely poor. Since improvement of prognosis and/or response to treatment by personalized and precision treatments requires earlier and precise diagnostic markers, discovery of prognostic markers attracts more attention. Apoptosis-inducing factor, mitochondrion-associated 3 (AIFM3) is highly expressed in several cancers including CCA. The present study investigated whether the serum AIFM3 level can be used as a potential marker for CCA prognosis. For this purpose, we first determined secretory protein nature of AIFM3 using bioinformatic tools. The results show that although AIFM3 lacks signal peptide, it can be secreted into plasma/serum via an unconventional pathway. Then, the AIFM3 levels in the sera of 141 CCA patients and 70 healthy controls (HC) were measured using a semi-quantitative dot blot assay. The results show that the AIFM3 level in the sera of CCA group was significantly higher than that of HC. When correlation between serum AIFM3 levels and the clinicopathological parameters of CCA patients were examined, serum AIFM3 levels correlated significantly with lymph node metastasis, age, and the patients’ overall survival (OS). Higher AIFM3 levels were significantly associated with shorter OS, and only AIFM3 was an independent prognostic marker for CCA. In conclusion, AIFM3 can be used as a prognostic marker for CCA.

## 1. Introduction

Cholangiocarcinoma (CCA) is a cancer originating from the bile duct epithelial cells (cholangiocytes). The incidence of CCA in Southeast Asia, especially in northeastern Thailand, is remarkably high in association with carcinogenic liver fluke, *Opisthorchis viverrini*, infection, which is known as the major risk factor for CCA in this area [1]. Related to this, almost all CCA patients in Thailand are of intrahepatic type as a silent and aggressive cancer so that prognosis of the patients is absolutely poor due to difficulties of early detection. In addition, CCA is generally resistant to chemotherapeutic drugs [2]. Currently, several serum markers such as carbohydrate antigen 19-9 (CA19-9), carcinoembryonic antigen (CEA), and mucin 5 subtype AC (MUC5AC) have been used as diagnostic markers of CCA. Although they are reliable markers for pancreatic or colorectal cancers, the sensitivity and specificity of those markers for CCA diagnosis are not quite satisfactory [3,4,5]. In addition, although CA19-9 and CEA have been used for clinical management of patients and prognostic prediction [6,7], its individually predictive value is not satisfactory even when the combination of those markers are used to predict prognosis for CCA. Therefore, it is critically necessary to discover novel markers to improve diagnosis and prognosis for CCA. 

Apoptosis-inducing factor, mitochondrion-associated 3 (AIFM3) is a mitochondrial protein/flavoenzyme and its gene is located on chromosome 22q11.21. Mature human AIFM3 protein consists of 598 amino acids with a molecular weight of 66 kDa and is predominantly localized in the mitochondria [8]. AIFM3 is involved in the induction of apoptosis of human embryonic kidney cells, HEK293 [8]. The predicted structures of human AIFM3 revealed two regions: iron-sulfur containing FeS-P Rieske domain and pyridine nucleotide-disulfide oxidoreductase domain (Pyr_redox). The Rieske domain is important to induce apoptosis of HEK293 cells, although the precise mechanisms of AIFM3 induced-apoptosis remains unclear. In contrast, the Pyr_redox domain did not contribute to the pro-apoptotic process, but it has 35% amino acid sequence similarities with the apoptosis-inducing factor (AIF) oxidoreductase domain [8]. At present, the function of Pyr_redox domain has not been clarified [8]. Although AIFM3 is widely expressed in many tissues, the physiological role of AIFM3 or its role in cancer has rarely been reported. Using mitochondrial proteome analysis, we reported that AIFM3 was overexpressed in CCA tissues in comparison to the corresponding adjacent non-cancerous tissues, showing that AIFM3 can be a potential target molecule for CCA chemotherapy [9]. Recently, Zheng et al. reported that AIFM3 was highly expressed in breast cancer tissues and might be a potential biomarker for predicting prognosis of breast cancer patients [10].

AIFM3 protein is distributed in mitochondria and cytosol, but mature AIFM3 protein lacks a signal peptide. Thus, whether it can be secreted in plasma/serum or its serum levels in health and disease remains unclear. The aims of this study are to determine the AIFM3 expression levels in the sera of CCA patients and controls and to evaluate whether it could be a potential diagnostic/prognostic biomarker of CCA. 

## 2. Materials and Methods 

### 2.1. Sample Size Calculation 

In the preliminary study, using a dot blot assay, the median and quartile deviation values of serum AIFM3 levels of 40 CCA and 20 HC sera were determined. The results showed the median and quartile deviation value of AIFM3 relative intensity in CCA and HC sera were 0.92 ± 1.41 and 0.42 ± 0.12, respectively. Then, using these values of two groups for sample size calculation, the minimum sample size necessary for comparison of the median between CCA group and HC group were determined as 133 samples, using a PS program version 3.1.2 [11].

### 2.2. Sera from CCA Patients and HC

Pre-operative serum samples from 141 CCA patients who were diagnosed as having intrahepatic CCA (median age ± quartile deviation, 60 ± 6.5 years; range 31–76 years) including 4 cases of paired sera of pre- and post-operative samples (4–10 months after surgery; median 5.5 months), were provided by the Cholangiocarcinoma Research Institute, Faculty of Medicine, Khon Kaen University, Thailand. Inclusion criteria were the patients being diagnosed as having intrahepatic CCA by clinico-pathological examinations. The patients were subjected to preoperative and postoperative care without receiving any chemotherapeutic treatment. Exclusion criteria were those who were diagnosed as extrahepatic CCA or hepatocellular carcinoma. The HC sera were the left-over of 70 healthy persons (median age ± quartile deviation, 41 ± 7 years; range 19–85 years) who received the annual health check-up in the Faculty of Associated Medical Science (AMS-KKU Excellence Laboratory), Khon Kaen University, Thailand, with generally healthy appearance and had normal biochemical tests for liver and kidney functions. All serum samples were kept at −80 °C until use. All subjects gave their informed consent for inclusion before they participated in the study. The study was conducted in accordance with the Declaration of Helsinki, and the protocol was approved by the Ethics Committee of Khon Kaen University, Thailand (HE611411).

### 2.3. Prediction of Secretory Proteins

Three bioinformatic softwares were used to predict secretory protein nature of AIFM3 molecule; (i) SignalP software V.4.0 server (Department of Bio and Health informatics, Technical University of Denmark, Lyngby, Denmark) to predict signal peptide cleavage site in the amino acid sequence using D-score >0.45 for the presence of a signal peptide within a protein sequence [12]; (ii) SecretomeP software V.2.0 server (Department of Bio and Health Informatics, Technical University of Denmark, Lyngby, Denmark) to predict proteins with a neural network score (NN score) >0.5 for confirmation of secretory proteins via an unconventional secretion pathway without a signal peptide [13]; and (iii) Plasma Proteome Database (PPD) Version 2014 (under the Human Proteome Organizations (HUPO)) to identify proteins present in serum or plasma. PPD is one of the largest resources on plasma proteins [14].

### 2.4. Western Blot Analysis

Fifty micrograms protein each of randomly selected 6 HC and 6 CCA serum samples were dissolved in sample buffer (10% sodium dodecylsulfate (SDS), 1 M Tris-HCl, pH 6.8), and boiled for 5 min. The samples were separated on 12.5% SDS-PAGE at 120 V for 3 h at 4 °C. The samples were loaded and run in parallel with standard molecular weight markers. After electrophoresis, proteins were transferred electrically onto PVDF membrane (GE Healthcare Life Sciences, Little Chalfont, UK) for 1 h at room temperature. The membrane was blocked with 5% skim milk in Tris-buffered saline with 0.1% Tween-20 (1X TBST, pH 7.4) for 1 h at room temperature. The membrane was then incubated with 1:1000 dilution of rabbit polyclonal antibody against human AIFM3 (Cat#orb74964, Biorbyt, Cambridge, UK) overnight at 4 °C. The membrane was washed with 1X TBST, incubated with 1: 10,000 dilution of horseradish peroxidase-conjugated goat anti-rabbit IgG secondary antibody for 1 h at room temperature, and washed with 1X TBST. Finally, peroxidase activity was detected as chemiluminescence using an ECL plus reagent (GE Healthcare Life Sciences, Little Chalfont, UK) and quantitatively analyzed using an Amersham imager 600. The CCA tissue lysate that showed high expression of AIFM3 in the previous study [9] was used as a positive control.

### 2.5. Dot Blot Assay and Data Acquisition

The nitrocellulose membrane (GE Healthcare Life Sciences, Little Chalfont, UK) was soaked in 1X TBST for 10 min before placed on the spotting machine. Each serum sample was diluted at 1:3 with normal saline (NSS) and 2 μL each was spotted onto the membrane using a Bio-Dot microfiltration apparatus (Bio-Rad Laboratories, Inc., Hercules, CA, USA). For each assay, the pooled CCA sera was used as a positive control. Then, the membrane was soaked in 5% skim-milk in 1X TBST for 1 h at room temperature to block nonspecific binding. The membrane was then incubated with 1:1000 dilution of the primary antibody (rabbit polyclonal antibody against human AIFM3) (Cat#orb74964, Biorbyt, Cambridge, UK) overnight at 4 °C. The membrane was washed with 1X TBST and then incubated with 1:10,000 dilution of horseradish peroxidase-conjugated goat anti-rabbit IgG secondary antibody for 1 h at room temperature, and washed with 1X TBST. The chemiluminescent signal was detected using enhanced chemiluminescence (ECL) plus reagent (GE Healthcare Life Sciences, Little Chalfont, UK) and quantified on an Amersham imager 600. Dot blot result (each spot) was computed for AIFM3 intensity by ImageJ software v.1.52d (National Institute of Health, Bethesda, MD, USA). The experiment was performed in triplicate. To prepare a standard curve, recombinant AIFM3 protein (Cat#H00150209-P01, Abnova, Taipei, Taiwan) with a known concentration (0.03 µg/µL) was diluted two fold as, 30, 15, 7.5, 3.75, 1.875, 0.9375, 0.4687, 0.2343, and 0.1171 ng/µL. The intensities of AIFM3 protein in the sera were normalized using AIFM3 intensity in a positive control as a relative expression [15,16]. Subsequently, relative expression of AIFM3 in each serum sample was calculated based on the standard curve prepared using the standard recombinant AIFM3 protein. 

### 2.6. Statistical Analysis

The data are presented as the median ± quartile deviation or the mean ± standard deviation with the range (minimum to maximum). The different values between two independent sample groups were estimated using the Mann–Whitney test. The associations between serum AIFM3 levels and patients’ clinicopathological parameters were analyzed using the Fisher’s exact test. The correlation between two variables was analyzed by the Spearman’s correlation test. In addition, comparison between preoperative and postoperative serum levels was tested using the paired t test. Cutoff Finder [17] was used to determine optimal cut-off to dichotomize the relative intensity of AIFM3 as low and high serum levels for clinicopathological parameters analysis using the method with the most significance (log-rank test) of correlation with survival variable. Briefly, data of serum AIFM3 level in CCA group, survival time, and status of patients were put into the Cutoff Finder, and defined variables as biomarker, survival time, and event, respectively. Then, “Survival:significance (log-rank test)” was selected as a method for cutoff determination. Overall survival curves were analyzed using the Kaplan–Meier method and log-rank test. The Cox proportional hazards regression model was used for univariate and multivariate analysis. *p* < 0.05 was considered as statistically significant. GraphPad Prism v.7 software (GraphPad Software Inc., La Jolla, CA, USA) and IBM SPSS v.16 software (IBM Corp., Armonk, NY, USA) were used for statistical analyses.

## 3. Results

### 3.1. Bioinformatic Analysis of AIFM3 Protein to Predict its Secretory Protein Nature

Using SignalP software, AIFM3 gave a score of 0.023, indicating that AIFM3 lacks a signal peptide and cleavage sites so that it is not a conventional secretory protein (ER-Golgi pathway). After SecretomeP software analysis, AIFM3 gave a score of 0.786, suggesting that it can be an unconventional secretory protein. However, neither AIFM3 has been listed in the PPD nor its serum/plasma level has been reported in the literature.

### 3.2. AIFM3 Expression in the Serum Samples

**Figure 1:**
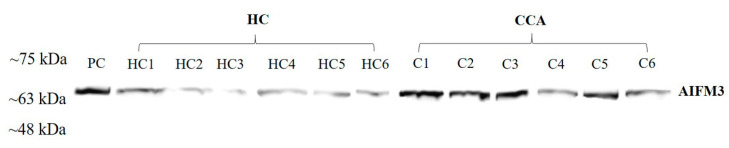
The validation of apoptosis-inducing factor, mitochondrion-associated 3 (AIFM3) expression in serum samples. AIFM3 was investigated by Western blot analysis; PC = positive control, HC = healthy control, C = CCA.

To investigate whether AIFM3 protein can be detected in the sera of CCA patients, the AIFM3 protein levels in the randomly selected sera from CCA and HC groups were examined using Western blot analysis. The results revealed the clear presence of approximately 66 kDa size band in all of the sera of both groups. Furthermore, the AIFM3 level in the sera of CCA patients appeared to be higher than that of HC (Figure 1).

### 3.3. Serum AIFM3 Levels of CCA and HC

**Figure 2:**
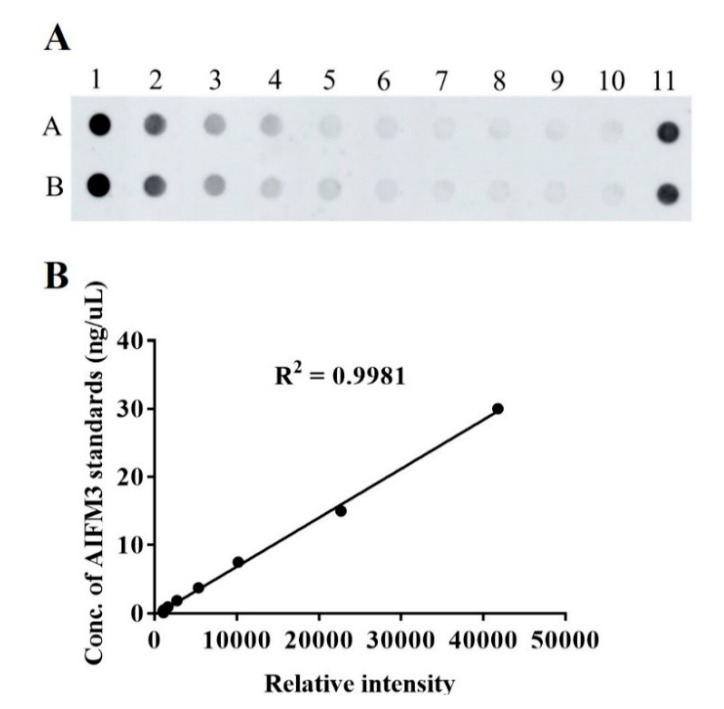
AIFM3 standard curve for dot blot assay. (**A**) The image of dot blot assay for AIFM3. Samples A1 to A9 were the serial 2-fold dilution of the AIFM3 standard protein, sample A10 was the blank control, sample A11 was positive control pooled sera, and row B was the duplicated row of A. (**B**) The standard curve of AIFM3 levels.

**Figure 3:**
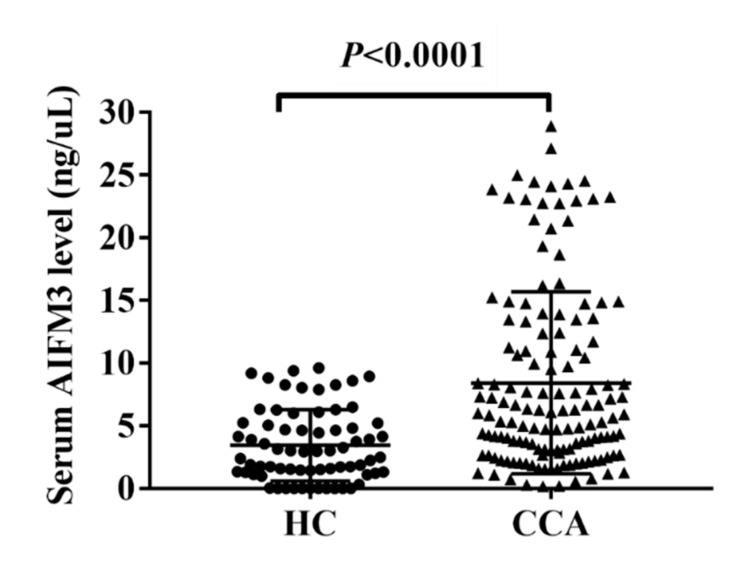
Serum AIFM3 levels of healthy control (HC) and cholangiocarcinoma (CCA) groups using dot blot assay. Long horizontal line: mean value, short upper, and lower lines: standard deviation range. The mean level ± standard deviation of AIFM3 was 3.258 ± 2.671 in HC group and 8.419 ± 7.269 in CCA patients group. * Significant difference of serum AIFM3 level between groups (*p* < 0.05).

A representative dot blot image is presented in Supplementary Data (Figure S1). The standard curve of AIFM3 is shown in Figure 2A,B. The demographic and clinical data of the participants are summarized in Table 1. AIFM3 levels of 141 serum samples from CCA patients and 70 from HC were measured quantitatively using a dot blot assay and the standard curve of AIFM3. As presented in Figure 3, the mean AIFM3 level of the sera of CCA patients was significantly higher than that of HC (*p* < 0.0001).

To ensure the reliability/reproducibility of the dot blot assay, serum samples were shuffled and randomly spotted onto the membrane (Figure S2A). When the results were compared with those of the results of the original experiment shown in the results, a linear correlation was observed between the first set and the second set of shuffled spotting (Figure S2B). To validate the accuracy of dot blot quantification, the correlation of the ratio of intensity between Western blot and dot blot was examined using three selected serum samples (high, medium, and low expression of AIFM3 in dot blot) of CCA patients. A linear correlation was observed between Western blot and dot blot (Figure S3).

### 3.4. Correlation between AIFM3 Expression in Serum and CCA Tissues

**Figure 4:**
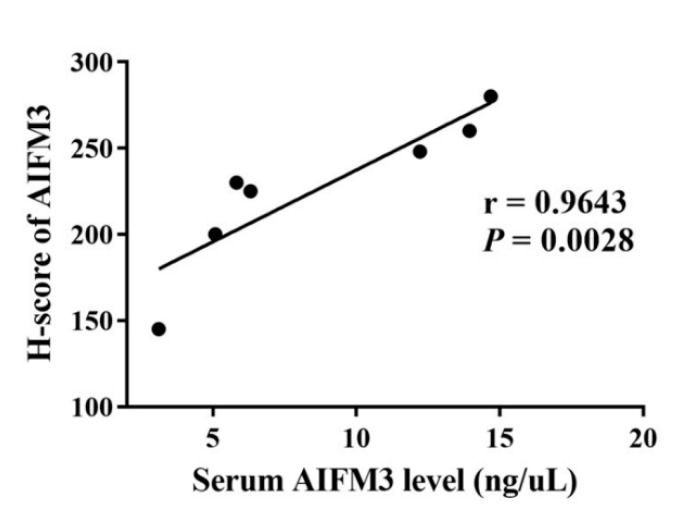
Validation of the accuracy of dot blot quantification. The correlation between H-score of IHC and serum AIFM3 level of the seven paired samples.

As presented in Figure 1, serum AIFM3 levels were notably low in the HC group and were markedly high in some patients with CCA. Thus, we examined whether AIFM3 produced and released from CCA cells is a major source of serum AIFM3. Among 141 serum samples of CCA patients, 7 cases have immunohistochemical staining of AIFM3 in CCA tissues in our previous research [9]. The graded staining intensity of those 7 specimens were from the previous study [9]. Then, correlation between the serum AIFM3 levels and AIFM3 expression in the corresponding CCA tissue was examined. The results show that serum AIFM3 levels were correlated with tissue AIFM3 expression, suggesting that serum AIFM3 is mainly derived from CCA tissues (Figure 4).

### 3.5. The Correlation of Serum AIFM3 Levels with Clinical Parameters

**Figure 5:**
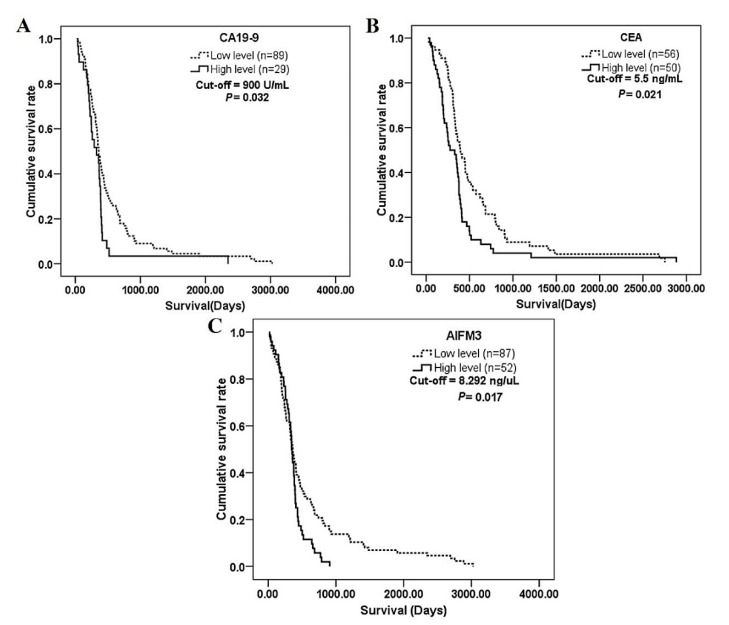
Correlation between serum AIFM3, carcinoembryonic antigen (CEA) and carbohydrate antigen 19-9 (CA19-9) levels and the survival of CCA patients. CCA patients were divided into high and low (**A**) serum CA19-9, (**B**) CEA, and (**C**) AIFM3 groups using the cut-off values of CA19-9 (900 U/mL), CEA (5.5 ng/mL), and AIFM3 (8.292 ng/uL), respectively using Cutoff Finder [17]. Kaplan–Meier curves showing overall survival (OS) of CCA patients having high (solid line) and low (dashed line) serum levels. Significant difference in the survival time was observed between high and low AIFM3 level groups (log-rank test *p*-value = 0.017).

To examine the possible clinical importance of AIFM3, Cutoff Finder [17] was used for determining optimal cut-off to dichotomize the AIFM3 levels as low and high serum levels. Then, CCA patients were divided into high serum AIFM3 and low serum AIFM3 groups, and the associations between serum AIFM3 levels and clinical parameters were analyzed. The results demonstrated that serum AIFM3 level was associated with lymph node metastasis, age, and survival time (*p* = 0.001, *p* = 0.002, and *p* = 0.017, respectively), but not with other parameters (Table 2). To elucidate the relationship between age and serum AIFM3, Spearman’s test revealed no correlation between age and serum AIFM3 levels both HC and CCA group (Figure S4), and we confirmed that serum AIFM3 level was not influenced by age. Potentially in association, the mean survival time was shorter for the high serum AIFM3 group compared with the low serum AIFM3 group. Correlation between serum AIFM3 level and the OS time was further confirmed using the Kaplan–Meier analysis. The OS time of CCA patients with high serum AIFM3 level was significantly shorter than that of CCA patients with low AIFM3 level (340 vs. 458 days; *p* = 0.017; Figure 5C). When the same analysis was performed using CEA and CA19-9, their serum level did not clearly segregate the survival time (Figure 5A,B). Furthermore, the multivariate Cox regression analysis demonstrated that only AIFM3 was an independent prognostic marker for CCA with hazard ratio of 3.15 (95% CI, 1.92–6.37) as shown in Table 3 and Table 4.

### 3.6. Evaluation of the Prognostic Potential of Serum AIFM3 in Comparison with other Prognostic Markers

To compare the prognostic potential of serum AIFM3 level and currently used markers, CEA and CA19-9, we performed Cox regression analysis for those 3 markers and the mortality (Figure 5, Table 4). The results showed that the serum AIFM3 level and CEA could be prognostic markers for CCA patients. Moreover, when the correlation between those 3 markers were examined (Figure S5), serum AIFM3 level did not correlate with serum CEA or CA19-9 levels. Thus, AIFM3 was identified as an independent prognostic marker for prognostic prediction in CCA patients.

**Figure 6:**
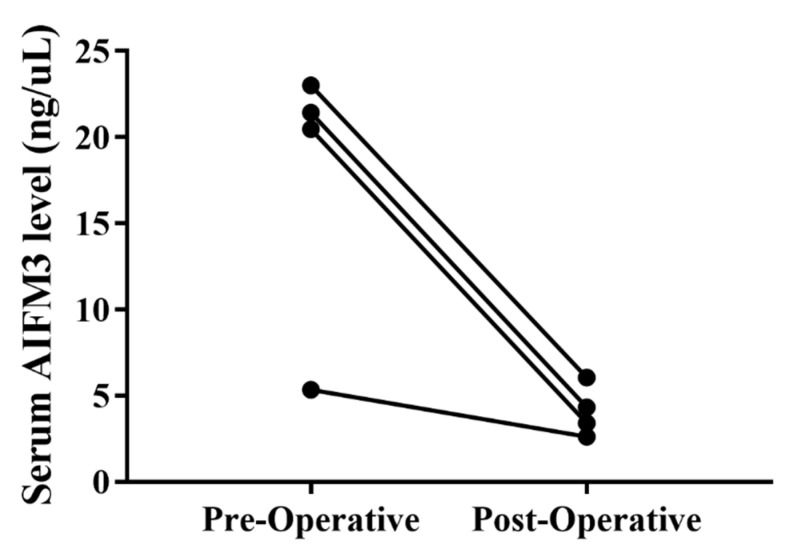
Comparison of serum AIFM3 level between preoperative and postoperative. All 4 CCA patients showed decrease of AIFM3 level after tumor resection.

To ensure whether AIFM3 can be a prognostic marker for CCA patients, although this is a preliminary study with small sample size, serum AIFM3 levels were measured for the paired pre- and post-operative sera of 4 CCA patients. As shown in Figure 6, serum AIFM3 level of all 4 CCA patients was significantly decreased (*p* = 0.032) after surgery. 

## 4. Discussion

We previously reported the over-expression of AIFM3 in CCA tissues [9]. AIFM3 is over-expressed also in breast cancer tissues. Thus, AIFM3 might be a potential biomarker for some cancers [10]. Nevertheless, the AIFM3 expression levels in the sera of patients with diseases including CCA is lacking. Nowadays, the circulating blood is a minimally invasive sample to discover new potential markers for diagnosis and prognosis of cancers [18]. In this study, SignalP, SecretomeP, and PPD were used to predict secretory protein nature of AIFM3. By bioinformatic analyses, AIFM3 is not listed in PPD, and it lacks a signal peptide predicted by SignalP. Still, SecretomeP results suggest that AIFM3 protein is assumed to be a secretory protein via an unconventional secretory pathway (ER/Golgi-independent pathway) [19]. Our results reported here is the first to measure AIFM3 levels in the sera of HC and patients with diseases.

Apart from AIFM3, several mitochondrial proteins such as mitochondrial ribosomal protein, mitochondrial stress 70 protein (mortalin), pyruvate dehydrogenase kinase 3, and manganese superoxide dismutase have been detected in the serum, and has been used as serum marker for breast cancer, colorectal cancer, CCA, and ovarian cancer [20,21,22,23] The present results suggest that AIFM3 should be added as a mitochondrial protein marker in the sera of patients with CCA and probably other cancers. In the present study, serum AIFM3 level in the CCA patients group was significantly (*p* < 0.0001) higher than that of HC. Additionally, serum AIFM3 levels were correlated with AIFM3 expression in the corresponding CCA tissues [9], suggesting that CCA cells are the major source of high AIFM3 level in the sera of CCA patients. To elucidate further, serum AIFM3 is mainly derived from CCA tissue, investigation of production/release of AIFM3 from CCA cells should be examined using CCA cell lines to ensure secretory protein nature of AIFM3. In addition, in this study, AIFM3 protein was detected in the majority of HC sera. Since AIFM3 is produced by a wide range of human cells/tissues but no known functions [9,10], AIFM3 might have an important role in systemic physiological regulation of human body.

In the present study, the estimation of OS by Kaplan–Meier was significantly shorter in patients with high serum AIFM3 level than in those with low serum AIFM3 level. In addition, in this study, AIFM3 was identified as an independent prognostic marker superior to CEA or CA19-9 for prognostic prediction of CCA patients. The concordant findings of high expression of AIFM3 in tumor tissue and poor OS have also been reported in breast cancer [10]. On the other hand, higher expression of AIFM3 mRNA dataset is correlated with greater patients’ survival time in bladder cancer tissues [24]. Besides, in this study, high serum AIFM3 level was associated with lymph node metastasis which was similar to the recent report in breast cancer tissues [10]. Remarkably, the higher AIFM3 level in CCA patients appeared to be divided into two groups. We speculated that the distribution pattern of the higher AIFM3 level might be involved in distant metastasis or clinical stage subgroups for predicting worse prognosis. Related to this, the AIFM3 gene is located on chromosome 22q11, a small segment of chromosome 22 which has been implicated in contributing to metastasis and progression of colorectal cancer, breast cancer, and prostate cancer [25]. Furthermore, the protein–protein interaction networks revealed that AIFM3 molecule linked to mitochondrial carcinogenesis-related proteins and various key molecules in cancer progression [9]. Since the high serum AIFM3 level is associated with the poor prognosis and lymph node metastasis of the patients with CCA, the role of AIFM3 in tumor invasion/metastasis as well as tumor proliferation requires examination in the future. 

In this study, to examine clinical applicability of AIFM3 protein for CCA prognosis, although it is a small scale preliminary study, we measured serum AIFM3 levels of pre- and post-operative paired sera of CCA patients after curative surgery. The results showed that serum AIFM3 levels were significantly decreased in all 4 cases after surgical removal of tumor mass. These results suggested that AIFM3 might be a useful prognostic marker for CCA. The ability of serum AIFM3 level for following-up CCA patients should be verified using a larger number of paired samples. 

## 5. Conclusions

In the present study, higher serum AIFM3 level was associated with lymph node metastasis and poor overall survival of the patients, so that serum AIFM3 level could be used as a prognostic marker for CCA patients. Although higher expression of AIFM3 was observed in some cancers, this study firstly reported that serum AIFM3 level can be a prognostic marker of CCA. Serum AIFM3 level should be further investigated in closely related diseases and other cancer patients in order to discriminate other diseases. The biological roles of AIFM3 in physiological and pathological state still remains unclear. Therefore, the role of AIFM3 protein in CCA needs to be investigated in the future.

## Figures and Tables

**Table 1:** Demographic and clinical characteristics of study cohort.

Parameter (Normal Range)	HC (*n* = 70)	CCA (*n* = 141)	*p*-Value
Age	41 ± 7	60 ± 6.5 ^a^	* <0.001
	(19–85)	(31–76)	
Total protein	NA	7.5 ± 0.5 ^b^	NA
(6.5–8.8 g/dL)		(4.6–10)	
Total bilirubin	NA	0.6 ± 0.8 ^b^	NA
(0.25–1.5)		(0.2–24.9)	
Direct bilirubin	NA	0.3 ± 0.6 ^b^	NA
(0–0.5 mg/dL)		(0–13.7)	
ALT	21 ± 6	38 ± 20 ^b^	* <0.001
(4–36 U/L)	(4–36)	(1–795)	
AST	20 ± 4	40 ± 18.7 ^b^	* <0.001
(12–32 U/L)	(6–34)	(4–1112)	
ALP	47 ± 9	168 ± 79 ^b^	* <0.001
(42–121 U/L)	(27–98)	(35–1068)	
Serum AIFM3 levels (ng/µL)	3.258 ± 2.671	8.419 ± 7.269 ^c^	* <0.001
	(0.001–9.602)	(0.175–28.907)	
CA19-9	NA	67.7 ± 435.8 ^d^	NA
(0–37 U/mL)		(0.6–1000)	
CEA	NA	5.1 ± 4.7 ^e^	NA
(0–2.5 ng/mL)		(0.9–917.6)	

Value showed median ± quartile deviation (min–max), ^a,b,c,d,e^ represented number of analyzed samples = 136, 131, 141, 106, and 118 respectively, NA = not analyzed, ALT = alanine transaminase, AST = aspartate transaminase, ALP = Alkaline phosphatase. The difference between and among groups were calculated by Mann–Whitney test. * Significant difference between HC and CCA of each clinical parameter. Serum AIFM3 level showed mean ± standard deviation (min–max).

**Table 2:** The association between serum AIFM3 levels and clinicopathological features of CCA patients.

Clinical Parameters	No.	Serum AIFM3 Levels (ng/µL)		
		≤8.292	>8.292	*p*-Value ^a^
Gender (*n* = 136)				
Male	91	62 (45.5%)	29 (21.3%)	0.701
Female	45	26 (19.1%)	19 (14.1%)	
Lymph node metastasis (*n* = 137)				
No	62	50 (36.4%)	12 (8.7%)	* 0.001
Yes	75	40 (29.1%)	35 (25.8%)	
Age (Years)	136	58.2 ± 9.5	63.2 ± 6.9	* 0.002 ^b^
		(*n* = 89)	(*n* = 42)	
Total protein (g/dL)	131	7.3 ± 1.1	7.7 ± 0.8	0.094
		(*n* = 89)	(*n* = 42)	
Total bilirubin (mg/dL)	131	2.1 ± 4.1	1.3 ± 1.7	0.250
		(*n* = 89)	(*n* = 42)	
Direct bilirubin (mg/dL)	131	1.4 ± 2.6	0.9 ± 1.6	0.395
		(*n* = 89)	(*n* = 42)	
ALT (U/L)	131	69.7 ± 112.4	45.7 ± 38.9	0.475
		(*n* = 89)	(*n* = 42)	
AST (U/L)	131	88.6 ± 174.1	49.8 ± 38.2	0.624
		(*n* = 89)	(*n* = 42)	
ALP (U/L)	131	247.3 ± 191.7	209.9 ± 200.7	0.067
		(*n* = 89)	(*n* = 42)	
Survival time (days)	139	579.8 ± 649.6	358.0 ± 185.4	* 0.017 ^b^
		(*n* = 87)	(*n* = 52)	

* Statistically significant correlation, Fisher exact test. ^a^ These variables were analyzed for serum AIFM3 level low and high groups (cut-off value at 8.292) ^b^ The different values among two groups were estimated using unpaired *t*-test (Data represent mean ± SD) Note; Total 141 samples were not completely analyzed due to lack of clinical information of patients.

**Table 3:** The Cox proportional hazards regression analysis of clinicopathological parameters and serum AIFM3 levels in CCA.

Clinicopathological Factors	Univariate Analysis		Multivariate Analysis	
	HR (95% CI)	*p*-Value	HR (95% CI)	*p*-Value
Lymph node metastasis	1.13 (0.84–1.95)	0.279	1.38 (0.96–2.14)	0.103
(non-metastasis or metastasis)				
Histological grading	1.10 (0.56–1.21)	0.567	0.99 (0.47–1.23)	0.721
(non-papillary or papillary)				
Gender (female or male)	1.20 (0.71–1.65)	0.731	1.35 (0.65–1.27)	0.610
Age (≤60 or >60 yr)	1.07 (0.88–2.01)	0.345	1.16 (0.61–2.21)	0.442
Total protein	0.84 (0.56–1.24)	0.239	0.95 (0.50–1.41)	0.357
(≤8.8 or >8.8 g/dL)				
Total bilirubin	1.09 (0.64–1.88)	0.704	1.42 (0.65–2.59)	0.562
(≤1.5 or >1.5 mg/dL)				
Direct bilirubin	0.96 (0.67–1.59)	0.847	0.78 (0.31–1.99)	0.694
(≤0.5 or >0.5 mg/dL)				
ALT (≤36 or >36 U/L)	1.22 (0.90–2.02)	0.265	1.44 (0.99–2.72)	0.331
AST (≤32 or >32 U/L)	1.28 (0.94–1.81)	0.546	1.07 (0.83–2.47)	0.664
ALP (≤121 or >121 U/L)	1.30 (0.84–1.45)	0.567	1.45 (0.97–2.49)	0.704
AIFM3 levels	1.98 (1.28–3.26)	* 0.015	3.15 (1.92–6.37)	* 0.009
(≤8.292 or >8.292 ng/µL)				

Abbreviations: HR = hazard ratio; CI = confidence interval. * Statistically significant.

**Table 4:** The Cox proportional hazards regression analysis of AIFM3, CA19-9, and CEA levels in CCA.

Tumor Markers	Univariate Analysis		Multivariate Analysis	
	HR (95% CI)	*p*-Value	HR (95% CI)	*p*-Value
AIFM3 (cut-off 8.292 ng/µL)	1.98 (1.28–3.26)	* 0.015	3.15 (1.92–6.37)	* 0.009
CA19-9 (cut-off 900 U/mL)	1.21 (0.61–2.50)	0.361	1.39 (0.81–2.41)	0.240
CEA (cut-off 5.5 ng/mL)	1.57 (1.02–2.40)	* 0.038	1.81 (0.90–3.25)	0.088

Abbreviations: HR = hazard ratio; CI = confidence interval. * Statistically significant.

## Supplementary Materials

The following are available online at https://www.mdpi.com/2218-273X/10/7/1021/s1, Figure S1: The representative dot blot images of AIFM3 levels, Figure S2: Shuffling and randomization of serum samples, Figure S3: Validation of the accuracy of dot blot quantification, Figure S4: Correlation between the serum AIFM3 level and the age of study subjects and Figure S5: The correlation between the levels of serum AIFM3 and CEA or CA19-9.
