# Supplementary material for: Apoptosis-Inducing Factor, Mitochondrion-Associated 3 (AIFM3) Protein Level in the Sera as a Prognostic Marker of Cholangiocarcinoma Patients
Source: Biomolecules. 2020 Jul 10;10(7):1021. doi: 10.3390/biom10071021 (PMC7408035; doi:10.3390/biom10071021)
Supplement: Supplementary file 1 [file biomolecules-10-01021-s001.pdf]

biomolecules

MDPI

Article

Apoptosis-inducing factor, mitochondrion-associated 3 (AIFM3) protein level in the sera as a prognostic marker of cholangiocarcinoma patients

Supplementary Materials:

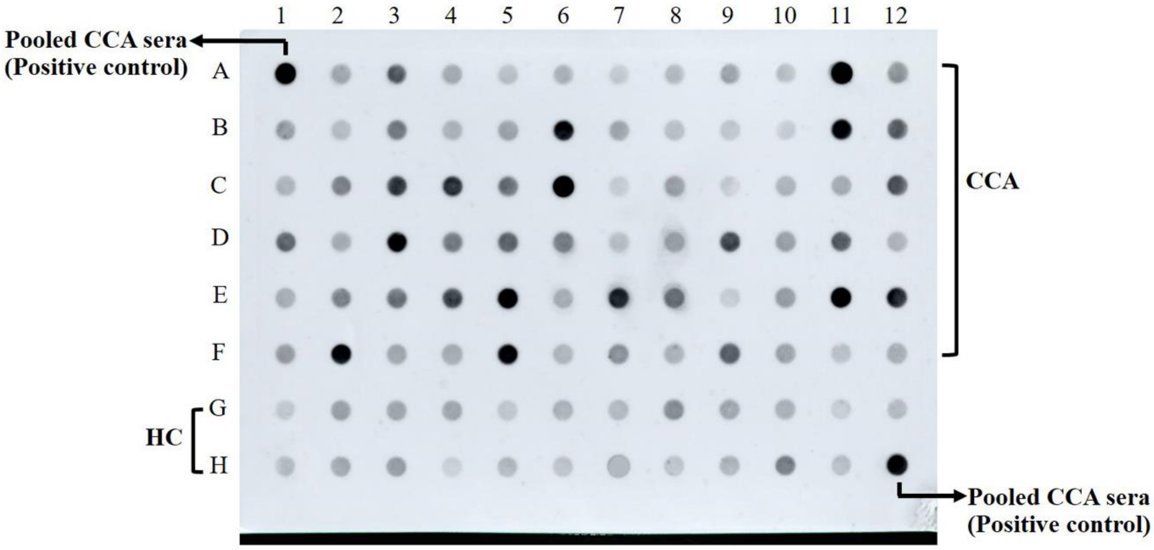

Figure S1 The representative dot blot images of AIFM3 levels from Amersham imager 600 analyzer. The spots were presented in order along the horizontal lines. A spot at the left upper corner and the right lower corner were a positive control (pooled CCA sera). The first six lines were CCA sera and the last two lines were HC serum samples.

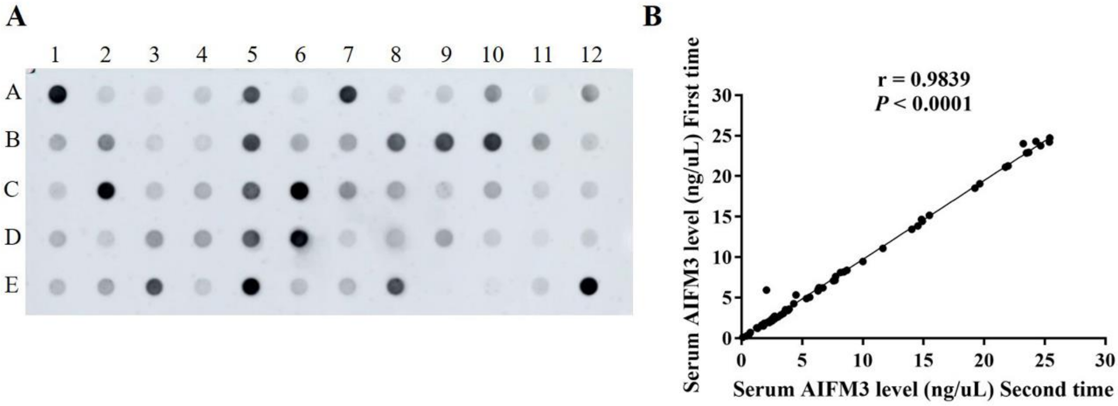

Figure S2 Shuffling and randomization of serum samples. (A) The results of dot blot. (B) The correlation of dot blot between first set and the second set of shuffled spotting.

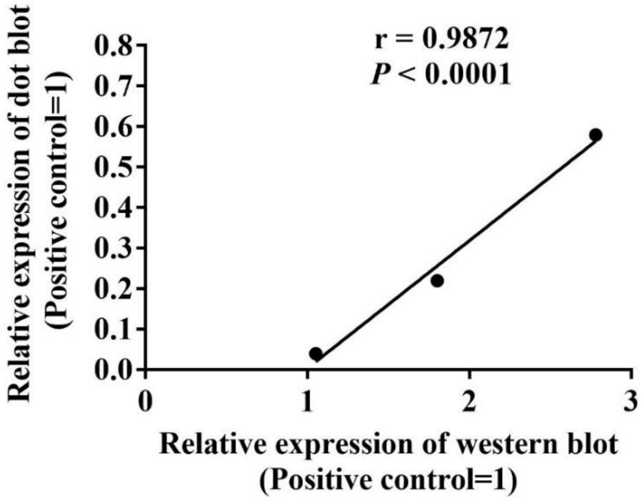

Figure S3 Validation of the accuracy of dot blot quantification. The correlation revealed positive correlation between western blot and dot blot assay with the relative expression of AIFM3 intensity/positive control

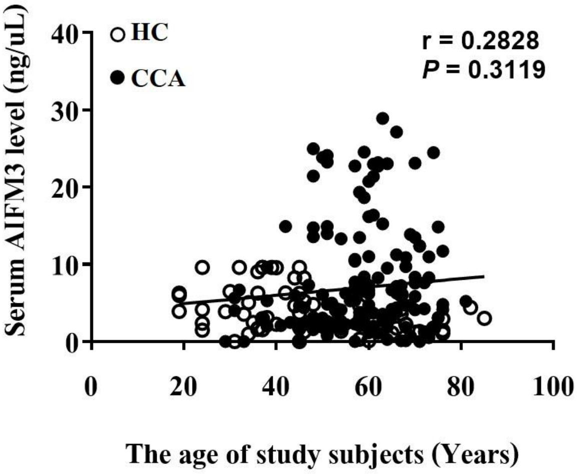

Figure S4 Correlation between the serum AIFM3 level and the age of the study subjects. The Spearman's correlation test showed serum AIFM3 level was not correlated with the age both HC and CCA group.

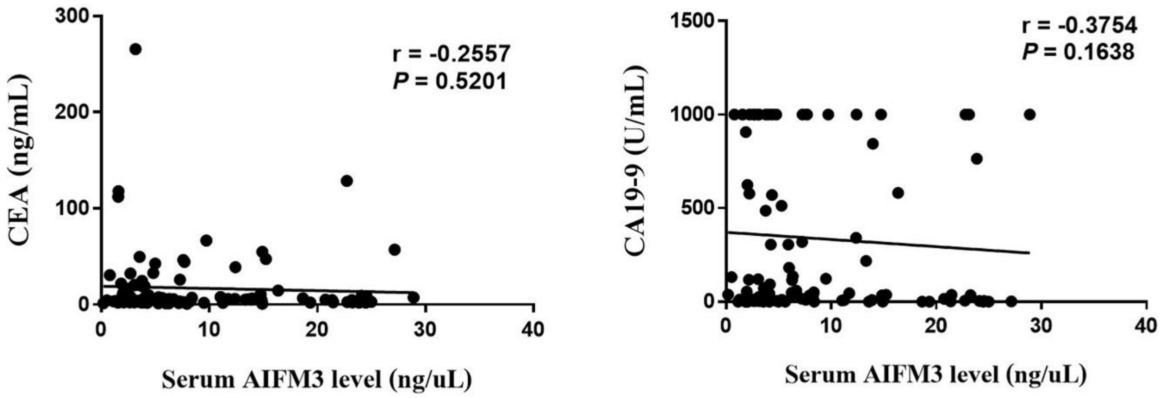

Figure S5. The Spearman's correlations tests between the levels of serum AIFM3 and CEA (panel A) and between the levels of serum AIFM3 and CA19-9 (panel B). The tests showed no correlations between the serum levels of AIFM3 and the levels of either CEA or CA19-9.
